# Evaluation of the Tooth Surface after Irradiation with Diode Laser Applied for Removal of Dental Microorganisms from Teeth of Patients with Gingivitis, Using X-ray Photoelectron (XPS) and Optical Profilometry (OP)

**DOI:** 10.3390/jcm11226840

**Published:** 2022-11-19

**Authors:** Iga Wawrzyk-Bochenek, Michał Łobacz, Sławomir Wilczyński, Mansur Rahnama, Justyna Szulc, Adam Konka, Anna Wawrzyk

**Affiliations:** 1 Department of Basic Biomedical Science, Faculty of Pharmaceutical Sciences in Sosnowiec, Medical University of Silesia, Kasztanowa 3, 41-205 Sosnowiec, Poland; 2 The Chair and Department of Oral Surgery, Medical University of Lublin, Chodźki 6, 20-093 Lublin, Poland; 3 Department of Environmental Biotechnology, Lodz University of Technology, Wólczańska 171/173, 90-530 Łódź, Poland; 4 Silesian Park of Medical Technology Kardio-Med Silesia in Zabrze, M. Curie Skłodowskiej 10C Str., 41-800 Zabrze, Poland

**Keywords:** tooth, diode laser, microorganisms, XPS, optical profilometry

## Abstract

Gingivitis is accompanied by microorganisms, including pathogens, which must be eliminated to speed up the treatment of inflammation. Laser irradiation may be one of the safe methods for reducing tissue contamination on the tooth surface. The aim of the study was the assessment of the tooth surface in patients with gingivitis after the use of a diode laser to eliminate microorganisms living there. In the first stage of the research, microorganisms were isolated (*Candida albicans*, *C. guilliermondii*, *Escherichia coli*, *Haemophilus parainfluenzae*, *Klebsiella oxytoca*, *Neisseria subflava*, *Rothia dentocariosa*, *Rothia mucilaginosa*, *Streptococcus pneumoniae*) from three patients with gingivitis, their identification confirmed using the MALDI-TOF MS technique (matrix-assisted laser desorption/ionisation time-of-flight mass spectrometry). Then, the irradiation process with a diode laser was optimized to a wavelength of 810 nm ± 10 nm in five variants to reduce microorganisms on the tooth. The tooth surface was analyzed by X-ray photoelectron spectroscopy (XPS) and optical profilometry (OP) before and after irradiation. 103 to 106 CFU were detected on a 0.4 cm^2^ tooth area. Nine types of bacteria and two types of fungi dominated among the microorganisms. The laser at the most effective biocidal dose of 25 W/15.000 Hz/10 µs, average = 3.84 W, with three uses after 15 s, increased the reduction of fungi from 57.97% to 93.80%, and bacteria from 30.67% to 100%. This dose also caused a decrease in the degree of oxidation and in the effect of smoothing on the treated surfaces.

## 1. Introduction

Dental plaque in the form of a biofilm consists of numerous bacteria, including over 400 species, that adhere permanently to the tooth surface [1]. Other reports indicate the presence of 700 species of prokaryotes [2], and total genomes across the oral cavity approaching 1500 [3]. The developing biofilm releases a variety of biologically active products, including lipopolysaccharides (endotoxins), chemotactic peptides, protein toxins, and organic acids. These molecules diffuse into the gingival epithelium, initiating a host response that ultimately leads to gingivitis and, under some circumstances, periodontitis [4]. The naturally occurring replacement of the mucosa epithelium three times a day prevents the accumulation of large masses of microorganisms, while surfaces that are not subject to exfoliation, e.g., teeth, dentures, or endosseous implants can accumulate thick biofilms. Established balanced biofilm maintains balance with the host. However, uncontrolled accumulation and metabolic activity of bacteria on hard surfaces, or external interference, are the main causes of inflammation including gingivitis [5]. Gingivitis is an inflammatory disease of the periodontal tissues. It is characterized by gum hyperemia, redness, bleeding, and swelling. The most common form of gingivitis is biofilm-induced gingivitis, caused by plaque bacteria. In many cases, untreated periodontal disease may be the cause of systemic disease processes, cardiovascular disease, stroke, or respiratory diseases, or may have adverse effects on pregnancy [6].

The elimination of microorganisms, especially pathogenic microorganisms, is of clinical importance and contributes to the improvement of human health. The traditional chemical methods used in dentistry to eradicate microorganisms are becoming less and less effective as microorganisms become opportunistic. This phenomenon applies especially to antibiotic therapy. Therefore, alternative methods of eliminating microorganisms are being sought. One such method is laser beam irradiation.

Lasers are increasingly used in dentistry and dental surgery as a method of decontamination and treatment of soft and hard tissues, because they can provide a large dose of energy per area unit [7]. The advantage of using the laser system is the reduction of microbial activity without vaporization and modification of the altered tooth tissues [8,9].

However, the generated heat can accumulate at a dangerous level during the emission of laser pulses, and this effect is the main limitation during irradiation [10].

Therefore, it is important to first establish the optimal laser performance parameters so that clinical laser decontamination procedures can be used. The effectiveness of the diode laser against microorganisms and the optimization of its work on abiotic surfaces in the oral cavity was confirmed by Wawrzyk et al. [11,12,13].

An important issue is the biocide of the laser, but in each case, the possibility of damaging the irradiated surface should be taken into account. The type of interaction between the laser and the material depends on the wavelength of the emitted radiation and the presence of radiation-absorbing chromophores in the material.

Ideally, a laser should be used, the radiation of which will be absorbed by microorganisms colonizing the surface, but not absorbed by the surface itself [14].

The degree of microbial reduction also depends on the smoothness of the surface. The rough surface contributes to the accumulation of dental plaque, and this favors the development of diseases in the oral cavity [15,16].

The tooth also has a different structure and roughness compared to other surfaces in the oral cavity. Human teeth have different surfaces depending on what structure they belong to (enamel crown, dentin, pulp, root, and periodontal ligaments) [17,18]. Additionally, the surface roughness of a nanohybrid composite is related to the type of finishing system [19,20]. 

The decontamination techniques used in dentistry should be biocidally effective, but should not adversely affect the structure and roughness of the surface [18]. 

Some techniques used in dentistry result in a very smooth surface, while others make the surface rougher. Research shows that each surface requires its own treatment method, e.g., polishing to obtain and maintain a smooth surface [16].

In order to assess the extent to which the laser affects the irradiated tooth surface, research techniques allow very accurate assessment of the changes taking place. Such techniques include X-ray photoelectron spectroscopy (XPS) and optical profilometry (OP).

XPS is a non-destructive technique for measuring the surfaces of solid materials, in particular chemical composition and electronic state. With this technique, it is possible to study the changes occurring at a very shallow depth of 3–5 nm. 

Optical profilometry is a method that facilitates the quantitative and qualitative assessment of changes on the surfaces of materials, it has been successfully used in clinical dentistry to assess changes in the surface of the teeth, especially in the second and third stages of erosion of tooth tissues [21].

The aim of this research was to evaluate the use of the diode laser for eliminating microorganisms isolated from the teeth of patients with gingivitis, in terms of its effect on the structure and chemical composition of the irradiated tooth surface. This study was conducted on the enamel surface because it is located near the soft tissues and is the main site of biofilm accumulation. The research hypothesis assumed that laser irradiation is an effective method of eliminating microorganisms from the tooth surface, with no negative effect on the structure of the tooth surface.

## 2. Materials and Methods

### 2.1. Patients and Materials

Swabs for microbiological tests were collected from teeth surfaces from 3 patients aged 18, 25, and 40 years, respectively. In the studied patients, the API (approximal plaque index) ranged from 71 to 80, indicating average oral hygiene. The PPD (ocket probing depth) was <4 mm, which means that they had no periodontitis. Meanwhile, BoP (bleeding on robing) was positive, i.e., bleeding was found during the examination. The oral hygiene index OHI (oral hygiene index) of the examined patients was on average 1, indicating poor oral hygiene. To optimize the work of the laser, the molar teeth removed from positions 38 and 48 were used. The extracted teeth were not occlusally useful and they were removed due to prophylactic indications. To assess the surface structure before and after irradiation, enamel surface of the third molar teeth without any traces of caries, removed from position 38, was selected.

### 2.2. Methods

#### 2.2.1. Assessment of Microbiological Contamination

In order to identify the microbiome inhabiting the surface of natural teeth, samples were taken from 3 patients (Section 2.1) who had teeth without signs of caries, but with symptoms of gingivitis. Individual 0.4 cm^2^ swabs were taken from the outer surface of the tooth enamel of each patient. The swabs were placed in sterile physiological saline (0.85% NaCl), shaken, and made into a stock. For quantitative testing, the suspensions from 10-fold dilutions were plated using the spread plate technique in triplicate on TSA (tryptic soy agar, Oxoid, UK) medium. The plates were incubated at 37 °C for 48 h. After incubation, the colony-forming units (CFU) were counted and the mean was calculated from triplicate replicates. The results are given as the mean number of microorganisms in CFU/0.4 cm^2^. For identification of microorganisms, 0.3 mL of the prepared suspension was plated on Columbia blood agar (Oxoid, UK) and incubated at 37 °C for 48 h in aerobic conditions with 5% CO_2_. Then, a pure culture on TSA broth was obtained from each of the grown colonies. The microorganisms were identified by MALDI-TOF MS (matrix-assisted laser desorption/ionisation time-of-flight mass spectrometry), using the Microflex LT system (Bruker Daltonics, Bremen, Germany) and the IVD HCCA matrix (Bruker, Billerica, MA, USA).

#### 2.2.2. Laser Irradiation

##### Laser Parameters

The samples were irradiated using an Elexxion claros (AG, Singen, Germany) laser with a fiber diameter of 600 µm at a wavelength of λ = 810 ± 10 nm in five variants as below:

- L1—decontaminate implant: 1 W/CW, average = 1.0 W, tip 600 μm, t = 1 min
- L2—expose implant: 15 W/15.000 Hz/10 µs, average = 2.30 W, tip 600 µm, t = 1 min
- L3a—periimplantitis surgical: 25 W/15.000 Hz/10 µs, average = 3.84 W, tip 600 µm, t = 1 × 15 s,
- L3b—periimplantitis surgical: 25 W/15.000 Hz/10 µs, average = 3.84 W, tip 600 µm t = 2 × 15 s,
- L3c—periimplantitis surgical: 25 W/15.000 Hz/10 µs, average = 3.84 W, tip 600 µm t = 3 × 15 s with 1 min cooling interval to eliminate overheating.

The sweep laser method was used in all samples.

##### Assessment of Decontamination Effectiveness on the Tooth Surface

To assess the biocidal effectiveness of the diode laser, seven strains of microorganisms isolated from the surface of the teeth were used. The most common and potentially pathogenic microorganisms were selected for study: *Candida guilliermondii*, *Candida albicans*, *Haemophilus parainfluenzae*, *Klebsiella oxytoca*, *Rothia dentocariosa*, *Rothia mucilaginosa*, and *Streptococcus pneumoniae*. Additionally, two standard strains from recognized culture collections (ATCC-American Type Culture Collection) were also used: *Escherichia coli* ATCC 13796 and *Staphylococcus aureus* ATCC 23235.

Individually, 50 µL samples of inoculum of microorganisms at concentrations of 10^6^ CFU/mL were applied in portions to the surfaces of the extracted teeth, waiting after each application until it was dry. Then, the tooth surface was irradiated with 5 different laser variants. After irradiation, rinsing was carried out, the stock of 10-fold dilutions was prepared, and the microorganisms were cultured according to the methodology described in Section 2.2.1. 

Experiments were carried out in triplicate for all nine strains (7 isolated from patients and 2 standard strains).

The reduction ratio (R%) was calculated according to the formula:

R% = ((N0 − N)/N0) × 100%,

where: 

N0—number of microorganisms before laser exposure, 

N—number of microorganisms after laser exposure.

#### 2.2.3. Statistical Analysis

For the number of microorganisms inhabiting the tooth surface, the mean value and standard deviation were recorded. To assess the significance of differences between the number of microorganisms on irradiated teeth and teeth without laser irradiation, one-way ANOVA was used and with significant difference at *p* < 0.05. The software STATISTICA 6.0 (Statsoft, Tulsa, OK, USA) was employed.

#### 2.2.4. Surface Morphology Analysis

To assess changes in the structure and morphology of the tooth surface, samples were selected that had been subjected to the L3 variant for three repetitions with 15 s of irradiation, because this was the most biocidally effective treatment.

##### X-ray Photoelectron Spectroscopy

The study of the chemical structure of the tooth surface before and after irradiation was performed in triplicate using X-ray photoelectron spectroscopy to monitor effectively even subtle changes in the elemental composition.

The samples of the supplied materials were analyzed in a multi-chamber UHV system (Prevac, Rogów, Poland) under high vacuum conditions. After placing the samples on the molybdenum support, the samples were degassed at room temperature to a constant high vacuum in the UHV system loading lock. Then, the carrier was transferred with the sample to the analytical chamber of the UHV system, and appropriate XPS analysis was performed. Monochromatic radiation from the Al anode was used as the excitation source, with the characteristic Al Kα line and energy of 1486.7 eV.

Survey spectra were applied to quantify the elemental composition of the surface of the tested materials. For the purposes of chemical analysis (functional groups), XPS spectra were recorded in a narrow energy range of electronic transitions (regions). Generally, XPS bands are characterized by low resolution, therefore the energy bands were deconvoluted (deconvolution method) into component peaks using the asymmetric Gauss–Lorentz function. Then, the sum of the components was programmatically adjusted to the spectral band. In order to normalize the spectroscopic measurements, the X axis of the spectra (binding energy, EB) was calibrated to the C1s aliphatic carbon peak, EB = 284.7 eV.

##### Optical Profilometry

The profilometric tests on the tooth surface were performed using the Contour GT-K1 optical profilometer by Veeco. The tests were carried out using the VSI (vertical scanning interferometry) technique.

Roughness measurements were performed at different locations on the tested sample surface for the L3 (a,b,c,) laser doses, for three scan sizes: 117.2 µm × 156.3 µm, 46.9 µm × 62.5 µm, and 1261 × 946 µm, giving the average roughness value, Ra. The images for the roughness measurement were flattened, the skew of the sample was corrected, and the waviness of the surface was taken into account.

The surface roughness measurement results are given with the expanded uncertainty (coverage factor k = 2). The roughness measurement uncertainty, Ra was estimated taking into account the de-calibration of the apparatus, recovery, test repeatability, and standard uncertainty. The uncertainty was estimated for the extreme points of the Ra measurement range (upper and lower limits) with the assumption of a trapezoidal distribution.

## 3. Results

### 3.1. Quantitative and Qualitative Analysis of Microbial Contamination of Teeth Surfaces

The mean microbial contamination in patients with gingivitis ranged from 10^3^ to 10^6^ CFU on the examined area (0.4 cm^2^) of the tooth. The highest number of microorganisms (4.50 × 10^6^ ± 0.36 × 10^6^ CFU/0.4 cm^2^) was detected for the first patient, lower in the second patient (1.44 × 10^4^ ± 0.61 × 10^4^ CFU/0.4 cm^2^), and the lowest in the third patient (3.58 × 10^3^ ± 0.45 × 10^3^ CFU/0.4 cm^2^). 

On the examined surfaces of the teeth, nine dominant microorganisms were detected and identified to species, including seven bacteria and two fungi (Table 1), which constituted over 95% of the cultured microorganisms. The remaining microorganisms were not identified per species, due to MALDI TOF method limitations. Among the microorganisms, Gram+ and Gram− bacteria were identified, including potential pathogenic species.

### 3.2. Evaluation of the Biocidal Effect of Laser Irradiation

The biocidal effect of a diode laser after the use of irradiation in various variants is presented in Table 2.

Under the influence of the diode laser, the reduction of the number of microorganisms on the teeth was at the level of 30.67–100%, depending on the irradiated microorganism and the applied dose. With the increase in laser power and repetition of irradiation, the reduction of microorganisms increased. A lower reduction was achieved for fungi and a slightly higher reduction for bacteria. The irradiation in the doses tested increased the reduction of fungi from 57.97% to 93.80%. Lower effectiveness was observed for the use of lasers on *Candida guillermondi* than on *Candida albicans*. Among the bacteria, *Klebsiella oxytoca* was the least reduced with the use of the L1 laser (t = 1 min), but the variant L3c (t = 3 × 15 s) eliminated 100% of this bacteria from the tooth surface. The L3c laser showed the highest efficiency in all cases of the tested microorganisms species, increasing with the number of repeats. The reduction after use of the L3 (a,b,c) laser ranged from 61.52% to 100%. Complete elimination of microorganisms under the influence of laser irradiation was achieved for *Klebsiella oxytoca* and *Rothia mucilaginosa* after using the L3 laser in three repetitions with a cooling break.

### 3.3. Surface Morphology Analysis

#### 3.3.1. The Chemical Structure of the Tooth Surface before and after Laser Irradiation

**Figure 1:**
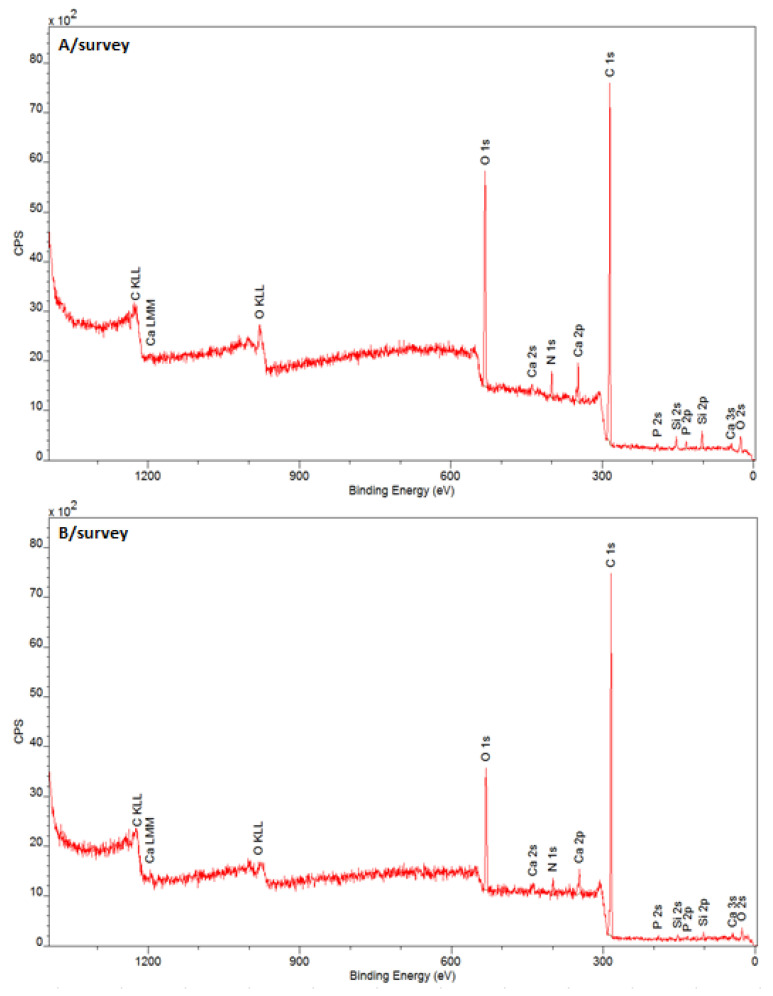
XPS survey of (**A**) unirradiated and (**B**) irradiated tooth surface, with laser variant L3c—25 W/15.000 Hz/10 µs, average = 3.84 W, tip 600 µm, t = 3 × 15 s.

The quantitative elemental composition of the tooth surface according to survey spectra is presented in Figure 1. 

It was shown that the presence of type I collagen was dominant in the outermost layer of enamel. This is evidenced by the presence of nitrogen atoms, mainly in the form of amide groups –N–(C=O), amino groups N–H, and carboxyl groups O–C=O amino acids. The presence of calcium Ca and phosphorus P atoms in the atomic ratio of Ca: P = 1.6 confirmed the presence of hydroxyapatite (HAp) in the deeper layer. The ratio of the number of carbon atoms to oxygen C:O in the control sample was K = 4.6:1, and in the lasered L = 7.8:1. Laser treatment increased the number of carbon atoms (mainly aliphatic) by more than 8%, and the C:O ratio increased 2.8 times after lasering. The measurement data indicated a strong decrease in the degree of oxidation of the tooth enamel surface after the laser operation.

The measurement data show that the increase in the amount of carbon comes at the expense of the oxidized forms of carbon, i.e., the groups C=O, C–OH, COOH, C–O–C, and CO_3_^2−^. Such changes suggest that laser radiation probably causes breakage of ester bridges, oxidation of C=O groups, and secondary condensation reactions of amino acids and carboxylic acids in the side chains of collagen fibers. The formation of peptide bonds may be evidenced by the increase in the number of amide groups (EB band = 400 eV) at the expense of amino groups (EB band = 399.26 eV).

The degree of oxidation of the enamel surface is expressed as the ratio of the total number of carbon atoms to the sum of the number of oxidized forms of carbon (O 1s band): K = 1.4:1 and L = 1.2:1. The obtained results confirm the fact that the degree of surface oxidation decreased after laser irradiation. The results show a decrease in the polarity of enamel and collagen. It should be emphasized that the measurements were made at two different locations on the enamel, therefore the differences in the results may also (at least in part) be caused by the heterogeneity of the chemical composition of the material.

Chemical analysis (functional groups) is presented in Table 3 in the form of XPS spectra recorded in a narrow range of electron transition energies (regions).

#### 3.3.2. Roughness of the Tooth Surface before and after Irradiation

In order to assess the tooth surface before and after laser L3 irradiation, surface microgeometry maps were constructed and the amplitude (height) roughness parameter for these areas was determined in relation to the reference plane. Shown as the arithmetic mean of profile ordinates (Ra), the test results are presented in Table 4.

The analysis of tooth enamel roughness depending on radiation dose showed that the average roughness in the smallest scanning areas of 47 µm × 62 µm did not change significantly after the laser irradiation process. In relation to the control, the average roughness of the enamel surface of the sample after L3a, t = 1 × 15 s increased for all scan sizes. Clear changes (reductions) in the surface roughness of the enamel in relation to the control sample were observed for the L3c sample, t = 3 × 15 s, for the scans 946 µm × 1261 µm and 117 µm × 156 µm. In the case of the scan 946 µm × 1261 µm, a 60% decrease in enamel roughness was noted, and in the case of the scan 117 µm × 156 µm, the average roughness decreased by 28%. In the case of the dose L3c, t = 3 × 15 s of laser radiation, a clear effect of smoothing on the enamel surface was observed.

**Figure 2:**
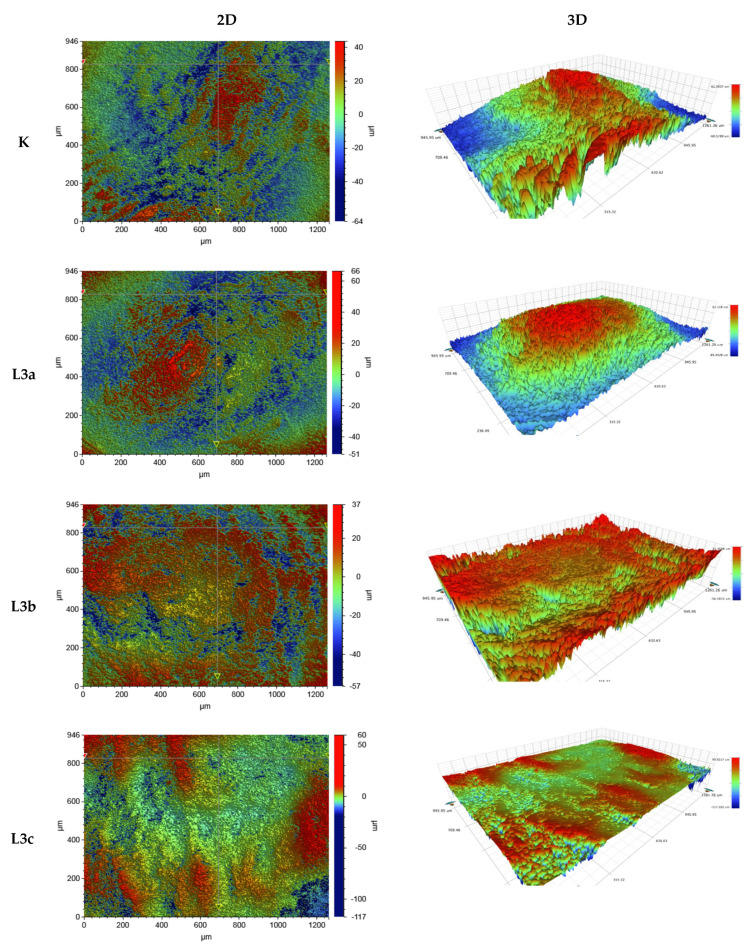
Sample 2D and 3D images (vertical and perspective views) of the topography and morphology of a fragment of the enamel surface, 946 µm × 1261 µm scan, before (K) and after laser irradiation (L3a–1 × 15 s, L3b–2 × 15 s, L3c–3 × 15 s).

Changes in the topography and morphology of the tooth enamel surface after application of L3c, t = 3 × 15 s laser radiation are presented as 2D and 3D images (Figure 2). 

## 4. Discussion

Some of the microorganisms identified in this study are included in the Human Oral Microbiome Database and are classified as normal oral flora [22]. The exceptions are *Streptococcus pneumoniae* and *Staphylococcus aureus*, which are nasal bacterial flora, and *Hemophilus parainfluense*, which is part of the natural flora of both the nose and mouth. Moreover, *Streptococcus pneumonie*, *Neisseria subflava* and *Rothia dentocariosa* were detected. These microorganisns may be pathogens that can cause disease in humans.

Among the microorganisms identified in these studies, numerous bacteria of the genus *Streptococcus* were found, which are the first to inhabit surfaces in the oral cavity and provide an “anchor” for the remaining microorganisms that make up the biofilm.

It is assumed that the cause of gingivitis is plaque in the form of a biofilm. *Streptococcus pneumonie* and *Haemophilus parainfluenzae* detected in these studies accompany the development of gingivalis [23].

Optical profilometry can be used to assess the degree of surface roughness before and after the physical application of disinfectants or decontamination agents. Optical profilometry is the research method for determining the surface characteristics of materials. This method enables the registration of three-dimensional images of the surface and the determination of metrological parameters [24]. Surface texture, especially its roughness and waviness, can be characterized by many parameters, the most important of which are Ra (arithmetic mean of the facade profile), Rq (mean square profile of the façade), Rp (height of the highest gable profile), and Ry (the lowest profile of recesses).

The method and timing of biofilm formation depends on the structure and morphology of the surface. Increased roughness provides better adhesion conditions for microorganisms. The surfaces in the oral cavity are characterized by different roughness. The value of Ra = 1.207 for the non-irradiated tooth (for the 117 × 156 µm scan) confirmed in these tests indicated that the tooth surface is rougher than porcelain (Ra = 0.452), zirconium (Ra = 0.337), titanium (Ra = 0.323), and differently polished composites (Ra = 0.578–0.696), as shown by Wawrzyk et al. [18,25].

The degree of roughness may change after laser disinfection is applied, and correct understanding of the various interactions of lasers with hard tooth tissues contributes to the improvement of restorative dentistry procedures [26].

Pejcic et al. proved that low-level laser irradiation can be used as an effective physical method of adjuvant treatment in conjunction with traditional periodontal therapy, and leads to better and longer-lasting therapeutic effects [27]. 

By assessing oxidative stress, Martu et al. proved that the combination of standard dental methods with a laser gave good clinical and paraclinical results [28].

The use of diode lasers for decontamination gives good results in various areas of life. However, its biocidal effectiveness depends primarily on the type of surface and its porosity. The effectiveness is lower on collagen or cellulose surfaces than on smoother surfaces such as composite, zircons, porcelain, or titanium, as proved by researchers [29,30,31,32].

As in those studies, reduction was obtained on the zirconium surface at the level of R = 99.08–99.87% for *S. pneumonie*, *N. subflava*, and *S. aureus*, and on the porcelain surface at the level of R = 84.57–99.94% for the same microorganisms [12,25].

In comparison, photoactivation therapy as an adjunct to periodontal treatment was effective in terms of clinical and microbiological parameters in patients with permanent dentures, especially in cases of severe periodontitis. Photoactivation therapy completely eradicated *Porphyromonas gingivalis* in patients with periodontitis, and *Tannerella forsythia* was reduced by 77.8% [33].

Dai and Gutknecht achieved a reduction in the number of microorganisms similar to these studies, using a diode laser for decontamination of *E. faecalis* and *E. coli* in root canals [34,35]. Meire achieved a 99.8% reduction in *E. faecalis*, also in the root canal [36]. Therefore, it is important to adjust the dose of laser irradiation to the type of surface on which it is applied in order to reduce microorganisms.

An important issue when carrying out surface decontamination is to minimize the influence of the disinfectant on the chemical properties that determine the surface structure and its susceptibility to biofilm deposition. In this study, the enamel subsurface layer was analyzed before and after laser irradiation with XPS.

X-ray photoelectron spectroscopy (XPS) is an effective analytical method for examining the elemental and chemical composition (type of functional groups and chemical bonds) of solid surfaces. In the high vacuum of the analyzer chamber, X-rays of sufficiently high kinetic energy knock out electrons (photoelectrons) from the cores of atoms at the surface layer of the tested sample. It is assumed that the penetration depth is on average 3–5 nm. The energy of the knocked-out electrons and their number depends on the atomic composition and the chemical state of the tested surface. The chemical environment of the atom (the type of chemical bonds between atoms, the degree of oxidation, and the vicinity of other atoms) induces changes in the binding energy (chemical shift) of the electrons with the atomic nucleus. The energetic separation of photoelectrons in the hemispherical detector generates the XPS spectrum, which, after appropriate treatment and analysis, provides qualitative and quantitative information on the chemical nature of the material’s surface. This method gives great opportunities to study a very thin subsurface layer, which is restricted by the limitations of other methods.

There are few scientific reports on the use of the XPS technique for examining the surface in the oral cavity, but so far it has been confirmed using this method that the tooth surface is composed of oxygen, carbon, nitrogen, calcium, and phosphor as its main elements, in accordance with earlier reports [37,38].

A study by Omae et al. showed that Er: YAG laser irradiation reduces the Ca/P ratio and denatures human dentin collagen [39].

In this study, the use of the XPS method proved that the tooth enamel was covered mostly with a layer of collagen, and the laser influenced the degree of oxidation of the tooth surface layer.

The decrease in the polarity of the enamel, or rather the collagen on its surface, may additionally act as a protective layer for the enamel and lower the solubility of the collagen layer. 

## 5. Conclusions

Diode laser applied at 25 W/15.000 Hz/10 µs, average = 3.84 W in 3 × 15 s effectively eliminated microorganisms from the tooth surface, while reducing its roughness. The proposed laser method, after verification on a wider group of microorganisms and finally under real conditions (irradiation of teeth in the patient’s mouth), can be proposed as an effective method to support gingivitis therapy.

## Figures and Tables

**Table 1:** The average number of microorganisms identified using the MALDI TOF technique on the surfaces of teeth.

No.	Microorganism	The Average Number on the Teeth [CFU/0.4 cm^2^]	Standard Deviation
1	*Candida albicans*	3.12 × 10^5^	2.55 × 10^4^
2	*Candida guilliermondii*	5.59 × 10^3^	1.41 × 10^1^
3	*Escherichia coli*	1.65 × 10^7^	2.12 × 10^6^
4	*Haemophilus parainfluenzae*	1.44 × 10^8^	9.05 × 10^6^
5	*Klebsiella oxytoca*	3.88 × 10^7^	2.26 × 10^6^
6	*Neisseria subflava*	1.11 × 10^6^	7.92 × 10^4^
7	*Rothia dentocariosa*	1.65 × 10^6^	9.05 × 10^4^
8	*Rothia mucilaginosa*	7.90 × 10^4^	4.24 × 10^3^
9	*Streptococcus pneumoniae*	1.77 × 10^5^	2.12 × 10^4^

**Table 2:** The results of the effectiveness of laser irradiation against the most common microorganisms on the teeth, with different numbers of repetitions, for non-irradiated samples. Laser doses: L1—1 W/CW, average = 1.0 W; L2—15 W/15.000 Hz/10 µs, average = 2.30 W; L3a,b,c—25 W/15.000 Hz/10 µs, average = 3.84 W.

Microorganisms	L1, t = 1 min	L2, t = 1 min	L3a, t = 1 × 15 s	L3b, t = 2 × 15 s	L3c, t = 3 × 15 s
	Reduction [%]				
*Candida albicans*	57.97 *	67.82 *	80.03	85.01 *	93.80 *
*Candida guilliermondii*	60.40 *	62.88 *	78.64 *	83.79 *	92.51 *
*Haemophilus parainfluenzae*	69.04 *	70.03 *	86.30 *	86.67 *	88.02 *
*Klebsiella oxytoca*	30.67 *	48.20 *	61.52 *	68.49 *	100.00 *
*Rothia dentocariosa*	68.97 *	72.43 *	88.35 *	92.90 *	98.77 *
*Rothia mucilaginosa*	78.09 *	80.76 *	91.19 *	96.37 *	100.00 *
*Streptococcus pneumoniae*	66.67 *	78.12 *	98.73 *	99.70 *	99.91 *
*Escherichia coli* ATCC 13796	69.35 *	74.56 *	78.67 *	94.70 *	99.30 *
*Staphylococcus aureus* ATCC 23235	65.08 *	70.07 *	85.18 *	97.17 *	99.99 *

* statistically significant difference in reference to the control sample; ANOVA and LSD at a significance level *p* < 0.05.

**Table 3:** Phase analysis of the tooth surface based on regional spectra. K (control, no irradiation) and L (after irradiation of L3c—25 W/15.000 Hz/10 µs, average = 3.84 W, tip 600 µm, t = 3 × 15 s).

Spectral Band	Band C 1s				
	K		L		Phase
	E_B_/eV	% At	E_B_/eV	% At	
C 1s A	284.11	10.2	284.10	11.9	C=C sp2
C 1s B	284.71	45.1	284.70	55.9	C–H
C 1s C	285.30	19.9	285.29	21.6	C–C sp3
C 1s D	286.11	8.5	286.10	2.9	C–OH
C 1s E	286.79	4.8	286.78	2.1	C–O–C
C 1s F	287.68	4.5	287.68	2.1	C=O
C 1s G	288.53	4.2	288.53	2.9	–O–C=O
C 1s H	289.30	3.0	289.30	0.5	CO_3_^2−^
**Spectral** **band**	**Band O 1s**				
	**K**		**L**		**Phase**
	**E_B_/eV**	**% At**	**E_B_/eV**	**% At**	
O 1s A	530.95	16.17	530.97	18.2	O–C=O
O 1s B	531.77	58.18	531.62	54.3	O=C
O 1s C	532.61	15.89	532.56	22.8	HO–C (C–(O)–C)
O 1s D	533.48	9.77	533.67	4.7	HO–C C–O–C
**Spectral** **band**	**Band N 1s**				
	**K**		**L**		**Phase**
	**E_B_/eV**	**% At**	**E_B_/eV**	**% At**	
N 1s A	399.30	19.9	399.26	8.1	C–NH_2_ (amina)
N 1s B	400.07	80.1	400.06	91.9	–N–(C=O) (amid)
**Spectral** **band**	**Band Si 2p**				
	**K**		**L**		**Phase**
	**E_B_/eV**	**% At**	**E_B_/eV**	**% At**	
Si 2p A	102.01	100.0	101.99	100.0	Si-O
**Spectral** **band**	**Band Ca 2p**				
	**K**		**L**		**Phase**
	**E_B_/eV**	**% At**	**E_B_/eV**	**% At**	
Ca 2p 3/2	347.44	50.7	347.43	50.7	Ca_3_PO_4_, CaCO_3_
Ca 2p 1/2	350.93	49.3	350.97	49.3	
**Spectral** **band**	**Band P 2p**				
	**K**		**L**		**Phase**
	**E_B_/eV**	**% At**	**E_B_/eV**	**% At**	
P 2p 1/2	133.93	49.5	133.70	49.5	PO_4_^3−^
P 2p 3/2	133.07	50.5	132.84	50.5	

**Table 4:** Roughness parameters determined for teeth before and after the laser irradiation process.

Roughness Parameter Ra (µm)				
Sample	K (No Exposure)	Tooth Surface after Irradiation L3a, t = 1 × 15 s	Tooth Surface after Irradiation L3b, t = 2 × 15 s	Tooth Surface after Irradiation L3c, t = 3 × 15 s
Measurement	946 µm × 1261 µm			
Average value	12.294	10.677	11.332	4.283
Measurement	117 µm × 156 µm			
Average value	1.207	0.551	0.481	0.396
Measurement	47 µm × 62 µm			
Average value	0.452	0.248	0.256	0.257

## Data Availability

Not applicable.
